# The opposite effects of fluvoxamine and sertraline in the treatment of psychotic major depression: a case report

**DOI:** 10.1186/1744-859X-9-23

**Published:** 2010-05-21

**Authors:** Akira Kishimoto, Ayako Todani, Junko Miura, Tetsuno Kitagaki, Kenji Hashimoto

**Affiliations:** 1 Yonago Medical Corporation, Yonago Clinic, Yonago, Tottori, Japan; 2 Yakumo Hospital, Matsue, Shimane, Japan; 3 Division of Clinical Neuroscience, Chiba University Center for Forensic Mental Health, Chiba, Japan

## Abstract

**Background:**

Psychotic major depression is a clinical subtype of major depressive disorder. A number of clinical studies have demonstrated the efficacy of the combination of an antidepressant (for example, a tricyclic antidepressant or selective serotonin reuptake inhibitor (SSRI)) and an atypical antipsychotic or electroconvulsive therapy (ECT) in treating psychotic major depression. In several studies, monotherapy of SSRIs such as fluvoxamine has been shown to be effective in the treatment of psychotic major depression.

**Methods:**

We report on a 36-year-old Japanese woman in whom fluvoxamine (a SSRI with sigma-1 receptor agonist) and sertraline (a SSRI with sigma-1 receptor antagonist) showed the opposite effects on psychotic symptoms in the treatment of psychotic major depression.

**Results:**

Symptoms of depression and psychosis in the patient who was non-respondent to antipsychotic drugs improved after fluvoxamine monotherapy. At 3 years later, a switch to sertraline from fluvoxamine dramatically worsened the psychotic symptoms in the patient. Then, a switch back to fluvoxamine from sertraline improved these symptoms 1 week after fluvoxamine treatment.

**Conclusion:**

Doctors should consider the monotherapy of sigma-1 receptor agonist fluvoxamine as an alternative approach to treating psychotic major depression.

## Background

Psychotic major depression is a clinical subtype of major depressive disorder and is characterized by psychosis accompanied by relatively severe depressive symptoms. Unfortunately, psychotic major depression frequently proves difficult to treat. A number of clinical studies have demonstrated the efficacy of the combination of an antidepressant (for example, a tricyclic antidepressant or selective serotonin reuptake inhibitor (SSRI)) and an atypical antipsychotic or electroconvulsive therapy (ECT) in treating psychotic major depression. In some cases, the clinician or patient may prefer to avoid antipsychotic drugs altogether because of the risk of extrapyramidal side effects in patients with psychotic major depression treated with these drugs [1-3].

SSRI fluvoxamine monotherapy has been reported to be effective against both the psychotic and depressive symptoms of this disorder [4-8], whereas sertraline appears to have a lower efficacy [9], suggesting that each SSRI might have a different spectrum of effectiveness in the treatment of psychotic major depression [10-12]. The precise mechanisms underlying the difference in efficacy for these two SSRIs are currently unknown. Here, we report a case in which fluvoxamine and sertraline showed marked opposite effects in the treatment of psychotic major depression.

### Case report

The patient was a 36-year-old Japanese woman who was diagnosed with psychotic major depression (F-32.3: severe depressive episode with psychotic symptoms) according to *International Classification of Disease, 10th edition *(ICD-10) criteria. The duration of her illness was approximately 15 years. Before admission to our clinic, she was treated with fluvoxamine (150 mg) and risperidone (4 mg) for about 1 year, and her symptoms were recovered. Then, she was admitted to the clinic due to delusions of persecution, including 'Everybody hopes that I return to the home from day care', 'The persons at day care are annoying me. I am troubling other persons', 'I do not like eyes of the persons who meet me at the return from day care. A man comes after me on the way of coming to the clinic', and depression. Risperidone (4 mg) was also added to the fluvoxamine (100 mg) treatment, but there no therapeutic effects were observed with respect to these delusions. The antipsychotic drug perospirone (12-24 mg) was then added, but it also showed no effect on the delusions. The fluvoxamine was therefore increased to 150 mg, and her delusions began to gradually decrease and finally disappeared completely. After recovery, risperidone treatment was stopped. Treatment with fluvoxamine (150 mg) monotherapy was maintained, and her condition remained good.

At 2 years after the disappearance of the delusions, the patient began overeating and oversleeping, as well as experiencing premenstrual syndrome. The *Diagnostic and Statistical Manual of Mental Disorders, fourth edition *(DSM-IV) criteria for atypical features include mood reactivity in addition to two of the following symptoms: overeating, oversleeping, severe fatigue or leaden paralysis, and a history of rejection sensitivity. Therefore, fluvoxamine (150 mg) was stopped because the diagnosis of atypical depression was doubted, and the patient was changed to another SSRI, sertraline (75 mg), as sertraline has been shown to be effective in the treatment of atypical depression and premenstrual syndrome [13-15]. At 3 days after the switch to sertraline, the patient suffered from several delusions such as 'Neighbors kick the wall of the house at home', 'They talk about me on the TV news', and 'The environment where I live now is bad'. In addition, she experienced cenestopathy such as 'I always think that there are chopsticks in the hole of my nose'. The sertraline was therefore stopped and was changed to fluvoxamine (150 mg). A week after fluvoxamine monotherapy treatment was given, the above symptoms and experiences rapidly disappeared.

## Discussion

Here we report a case showing the opposite effects of fluvoxamine and sertraline in a female patient with psychotic major depression that was non-respondent to antipsychotics. In this case, fluvoxamine monotherapy was effective in the treatment of psychotic major depression, and the switch to sertraline worsened the psychotic symptoms in the patient. The mechanisms underlying the opposite effects of these two SSRIs on the symptoms of a patient with psychotic major depression are currently unclear. This case suggests that fluvoxamine is clinically effective in the treatment of psychotic major depression, but sertraline may not be clinically useful for psychotic major depression although a further detailed study is necessary. It is thus likely that doctors should treat very carefully for the treatment of psychotic major depression using SSRI monotherapy.

One possible mechanism may be due to the difference in the action of these SSRIs at the endoplasmic reticulum protein sigma-1 receptors. Several pieces of evidence suggest that sigma-1 receptors play a role in the pathophysiology of major depression and in the active mechanisms of some antidepressants [16-20]. The inhibition constants (Ki) of fluvoxamine and sertraline at sigma-1 receptors are 36 nM and 57 nM, respectively [21]. Some recent studies have suggested that fluvoxamine is a potent agonist at sigma-1 receptors, whereas sertraline may be a sigma-1 receptor antagonist [18,19,22-24]. A positron emission tomography study demonstrated that fluvoxamine binds to sigma-1 receptors in the intact human brain at therapeutic doses [25], suggesting that sigma-1 receptors are involved in the active mechanisms of fluvoxamine [19]. Interestingly, the addition of haloperidol (a potent sigma-1 receptor antagonist) has been found to reduce the response rate to fluvoxamine from 69% to 45%, suggesting that haloperidol might antagonize the sigma-1 receptor activity of fluvoxamine [26]. Based on all these findings, it has been hypothesized that the sigma-1 receptors may be implicated in the efficacy of fluvoxamine for psychotic depression [7,8,10-12]. It seems that serotonin reuptake inhibition as well as sigma-1 receptor agonism may be involved in the active mechanism of fluvoxamine, since paroxetine (no affinity at sigma-1 receptors) has a lesser effect in psychotic major depression [27]. Although this case study does not clarify whether sigma-1 receptors are involved in the active mechanism of fluvoxamine, it is likely that the difference in the pharmacological actions (agonist or antagonist) of the two SSRIs at the sigma-1 receptors may have been related to the mechanisms of the opposite effects of the two SSRIs in this case. Thus, it seems that the antagonism of sigma-1 receptors by sertraline may be involved in the recurrence of psychotic symptoms in this case, although a further detailed study is necessary.

Another possibility is due to the potent inhibition (Ki = 22 nM for sertraline, Ki = 16,790 nM for fluvoxamine) of dopamine transporter by sertraline [28]. It is suggested that the relative potency of sertraline for dopamine transporter inhibition might differentiate its psychopharmacology from that of other SSRIs [29]. It is therefore possible that activation of the dopaminergic system by the inhibition of dopamine transporter may be involved in the mechanism of unwanted effects (deterioration of psychosis) of sertarline in this case, although further study is necessary.

## Conclusions

This case suggests that the sigma-1 receptor agonist fluvoxamine could be an alternative approach in treating psychotic major depression. More detailed randomized, double-blind studies should be performed to clarify the role of sigma-1 receptors in the efficacy of fluvoxamine for psychotic major depression.

## Consent

The treatment of the reported case was made according to standard clinical practice; however written informed consent was obtained from the patient for publication of this case report.

## Competing interests

The authors declare that they have no competing interests.

## Authors' contributions

AK, AT, JM and TK contributed to the clinical and rating evaluations during the follow-up periods. KH conceived of the study and participated in its study and coordination. All authors read and approved the final manuscript.
